# Local Persistence of Novel MRSA Lineage after Hospital Ward Outbreak, Cambridge, UK, 2011–2013

**DOI:** 10.3201/eid2209.151100

**Published:** 2016-09

**Authors:** Michelle S. Toleman, Sandra Reuter, Francesc Coll, Ewan M. Harrison, Sharon J. Peacock

**Affiliations:** University of Cambridge Department of Medicine, Addenbrooke’s Hospital, Cambridge, UK

**Keywords:** Bacteria, MRSA, staphylococci, Staphylococcus aureus, infection control, nosocomial, Cambridge, United Kingdom

**To the Editor:** Previously, we reported the use of whole-genome sequencing to investigate a putative methicillin-resistant *Staphylococcus* *aureus* (MRSA) outbreak in 2011 in the special care baby unit (SCBU) at the Cambridge University Hospitals National Health Service Foundation Trust (CUH) in the United Kingdom (*1*). The report identified 26 related cases of infection with or asymptomatic carriage of MRSA and showed that transmission occurred within the SCBU, between mothers on a postnatal ward, and in the community; the outbreak apparently resolved at the end of 2011. The outbreak strain, sequence type (ST) 2371, was of a novel multilocus ST related to the dominant hospital-associated lineage in the UK (ST22, EMRSA-15), but unlike most ST22 strains, this strain was Panton-Valentine leucocidin–positive (*2*). Since then, ST2371 has been identified as a prevalent community-associated MRSA clone in Southern India, and sporadic isolates have also been detected by whole-genome sequencing of MRSA in Denmark (*3*–*5*).

During April 2012–April 2013, we implemented genomic surveillance of MRSA isolated at the diagnostic microbiology laboratory at the CUH (F. Coll, unpub. data). From this, we noted that 10 isolates cultured from samples submitted from general practice (n = 7) and hospital wards (n = 3) during June 2012–February 2013 were classified as ST2371. Phylogenetic comparison between these 10 isolates and the 45 isolates from the original outbreak demonstrated that these strains were highly related (staphylococcal cassette chromosome *mec* IVc, Panton-Valentine leucocidin–positive, staphylococcal protein A [*spa*] type 852) (Figure).

**Figure:**
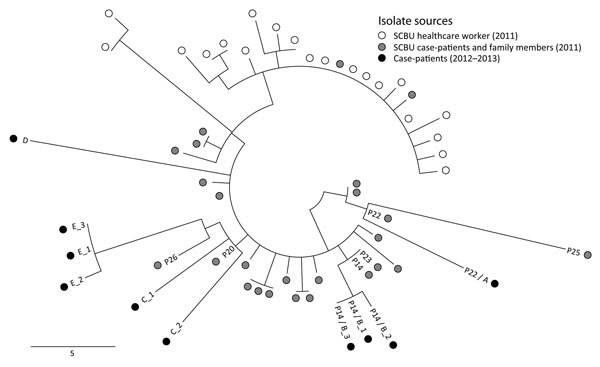
Midpoint-rooted phylogenetic tree based on single-nucleotide polymorphisms (SNPs) in the core genome of methicillin-resistant *Staphylococcus* *aureus* isolates from 2 investigations in the United Kingdom in 2011 and 2012–2013. Isolates were mapped against the EMRSA-15 reference genome. Open circles denote 20 individual colonies from a nasal swab culture taken from a healthcare worker during an outbreak in a hospital special care baby unit (SCBU) in 2011. Gray shaded circles denote isolates from 25 patients and their family members investigated during the 2011 outbreak. Black circles denote 10 isolates from 5 persons (case-patients A–E) from whom microbiological samples were taken during the 2012–2013 study. Numbers prefixed by P indicate the original study number used for each case during the 2011 outbreak investigation. Multiple samples from the same patient are identified by an underscore followed by the sample number. Two case-patients (P22/A and P14/B) were included in both outbreaks. Scale bar indicates SNPs.

We undertook an epidemiologic investigation to determine whether links could be identified between these new cases and the original outbreak. The 10 isolates were cultured from 5 patients (case-patients A–E), all of whom had a direct or indirect link to the 2011 outbreak. Case-patients from the 2011 outbreak are identified by the alphanumeric code assigned during that outbreak investigation (e.g., P22) (*1*). 

Case-patients A and B were also case-patients in the original SCBU outbreak (P22 on the postnatal ward and P14 in the SCBU, respectively). Case-patient C was born at the CUH and was not screened for MRSA, but both parents were case-patients in the SCBU outbreak (P20 and P26). Case-patient D was born at the CUH and discharged when 5 days old, which was 2 days before the birth of the presumed index case-patient of the original SCBU outbreak. The sample for the first isolate from case-patient D was collected almost 2 years later; acquisition could have occurred at the CUH or from subsequent contact with unsuspected carriers in the case-patient’s family or the community. On the basis of a matching surname, case-patient E was determined to likely be a member of the same family as case-patients P20, P26, and C. Soft tissue infection was documented in all 5 case-patients, supporting the original observation that ST2371 is associated with disease. Evidence of familial transmission in the original outbreak is further supported by transmission between case-patients P20, P26, C, and E. Furthermore, 2 case-patients infected during the original outbreak, P22/A and P14/B, continued to experience disease signs and symptoms for >15 months after their initial diagnosis.

Our data highlight the role of hospitals as reservoirs of MRSA and subsequent failure to track the entry and spread of MRSA in the community. MRSA decolonization was advised in all cases in the original outbreak, but this process clearly proved ineffective for case-patients A and B. Potential explanations include not implementing or completing the course of decolonization; failed decolonization; or limiting decolonization to only some members of an affected family. Although the outbreak in the hospital ward was resolved, the lack of a systematic surveillance program to monitor the incidence of noninvasive MRSA infections among the case-patients’ contacts and the community allowed this novel lineage to continue to cause disease in a group of linked persons. Considering recommendations to move from universal to targeted MRSA screening in hospitals in England (*6*), more active surveillance of any identified case-patients or carriers of MRSA in the community may be warranted.
